# Assessment of Early Tumor Response to Cytotoxic Chemotherapy with Dynamic Contrast-Enhanced Ultrasound in Human Breast Cancer Xenografts

**DOI:** 10.1371/journal.pone.0058274

**Published:** 2013-03-01

**Authors:** Jian-Wei Wang, Wei Zheng, Ji-Bin Liu, Yao Chen, Long-Hui Cao, Rong-Zhen Luo, An-Hua Li, Jian-Hua Zhou

**Affiliations:** 1 State Key Laboratory of Oncology in South China, Department of Ultrasound, Sun Yat-Sen University Cancer Center, Guangzhou, People’s Republic of China; 2 Department of Radiology, Thomas Jefferson University, Philadelphia, Pennsylvania, United States of America; 3 State Key Laboratory of Oncology in South China, Department of Anesthesiology, Sun Yat-Sen University Cancer Center, Guangzhou, People’s Republic of China; 4 State Key Laboratory of Oncology in South China, Department of Pathology, Sun Yat-Sen University Cancer Center, Guangzhou, People’s Republic of China; European Institute of Oncology, Italy

## Abstract

There is a strong need to assess early tumor response to chemotherapy in order to avoid adverse effects from unnecessary chemotherapy and allow early transition to second-line therapy. This study was to quantify tumor perfusion changes with dynamic contrast-enhanced ultrasound (CEUS) in the evaluation of early tumor response to cytotoxic chemotherapy. Sixty nude mice bearing with MCF-7 breast cancer were administrated with either adriamycin or sterile saline. CEUS was performed on days 0, 2, 4 and 6 of the treatment, in which time-signal intensity (SI) curves were obtained from the intratumoral and depth-matched liver parenchyma. Four perfusion parameters including peak enhancement (PE), area under the curve of wash-in (WiAUC), wash-in rate (WiR) and wash-in perfusion index (WiPI) were calculated from perfusion curves and normalized with respect to perfusion of adjacent liver parenchyma. Histopathological analysis was conducted to evaluate tumor perfusion, tumor cell density, microvascular density (MVD) and proliferating cell density. Significant decreases of tumor normalized perfusion parameters (i.e., nPE, nWiAUC, nWiR and nWiPI) were noticed between adriamycin-treated and control groups (*P*<0.01) 2 days after therapy. There were significant differences of tumor volumes between control and treated groups on day 6 (*P*<0.001) while there were no significant differences in tumor volume on days 0, 2 and 4 (*P*>0.05). Significant decreases of tumor perfusion, tumor cell density, MVD and proliferating cell density were seen in adrianycin-treated group 2 days after therapy when compared to control group (*P*<0.001). Dynamic CEUS for quantification of tumor perfusion could be used for early detection of cancer response to cytotoxic chemotherapy prior to notable tumor shrinkage.

## Introduction

Cancer treatment is a great challenge for medical research and clinical practice. Currently, chemotherapy is still one of the most important methods for cancer treatment, in which more than half of all patients with malignant tumors receive chemotherapy. Due to the fact that most of the malignant tumors are widely heterogeneous, the response to cytotoxic chemotherapy is variable even for the tumor with same pathologic types and differentiation grades. Therefore, it is important to precisely assess the response to cytotoxic therapy at its early time point, so as to reduce side effects and unnecessary costs of ineffective therapy [1].

The criteria most commonly used to assess the effectiveness of chemotherapy is the Response Evaluation Criteria in Solid Tumor(RECIST)which have become widely applicable in clinical oncological trials since it was published in 2000 [2]. Although it is useful protocol for estimating tumor response to chemotherapy based on morphologic changes, the RECIST has some disadvantages and limitations by using morphologic imaging methods. First, the tumor morphology changes on imaging might occur several weeks later regardless of a positive functional response to treatment [3], which could substantially delay the judgment of chemotherapy effectiveness. Second, conventional morphologic imaging could not differentiate tumor necrosis or fibrosis from residual tumor tissue [3], which could provide nonspecific information for assessing chemotherapy. Furthermore, newly developed chemotherapy (i.e., antiangiogenetic drugs) aims to ablate tumor neovascularities, which requires the assessment of tumor blood flow changes instead of tumor morphologic changes. Thus, it is critical to develop functional imaging techniques to better monitor tumor response to cytotoxic chemotherapy, which overcome the limitation of current criteria based on measurement of tumor size [1].

Functional imaging techniques such as positron-emission tomography (PET), dynamic contrast-enhanced magnetic resonance imaging (DCE-MRI), dynamic contrast-enhanced computed tomography (DCE-CT) and ultrasound (US) have been investigated to assess tumor response to chemotherapy by depicting the reductions in metabolic activity or the blood flow perfusion of the tumor [4]–[7]. Of these, US is an attractive modality for assessment tumor response to therapy because of the ease with which it can be repeated without exposing the patient or animal to any risk of radiation. Ultrasound imaging systems are also relatively inexpensive and mobile, a particular benefit for animal studies. Contrast-enhanced ultrasound (CEUS) offers the major advantages of evaluating tumor perfusion in real time and minimal invasiveness when compared to other methods for assessment of tumor perfusion. Quantification of tumor perfusion with CEUS has been studied to assess tumor response to antiangiogenic therapy, and preliminary results were promising [8]–[11]. Other than these molecular target drugs, currently most of the cancer patents still undergo the treatment of cytotoxic chemotherapy. We have previously shown that tumor blood flow perfusion changes after cisplatin treatment with CEUS [12]. However, to the best of our knowledge, the value of quantifying tumor blood flow perfusion with CEUS has not been tested in early assessing tumor response to cytotoxic chemotherapy. The purpose of this study was to determine whether CEUS can quantitatively identify blood flow perfusion changes for the evaluation of tumor response to a cytotoxic chemotherapeutic drug, adriamycin, in its early stage of the treatment.

## Materials and Methods

### Ethics Statement

This study was carried out in strict accordance with the recommendations in the Guide for the Care and Use of Laboratory Animals of the National Institutes of Health. The protocol was approved by the Committee on the Ethics of Animal Experiments of the Sun Yat-Sen University (Permit Number: 2012-A009). All surgery was performed under sodium pentobarbital anesthesia, and all efforts were made to minimize suffering.

### Cells

Human breast cancer cell line MCF-7 was obtained from the state key laboratory of oncology in southern China. MCF-7 cells were grown in DMEN culture medium (Hyclone Co., UT, USA) supplemented with 10% fetal bovine serum (Gibco, NY, USA), penicillin (50 U/mL), and streptomycin (50 µg/mL) at 37°C in a humidified 5% CO^2^ atmosphere.

### Animal Model

For inoculation, approximately 7×10^7^ MCF-7 cells suspended in phosphate-buffered saline were injected subcutaneously into the right flanks (at the level of the liver) of BALB/c nude female mice. They were 8-week-old with body weigh from 18 to 20 g.

### Imaging Protocol

Ultrasound imaging was obtained using a Sequoia 512 ultrasound unit associated with a 15L8 7.0∼14.0 MHz linear array transducer (Siemens, Mountain View, CA, USA). The probe offers a lateral resolution of 0.35 mm and an axial resolution of 10.25 mm (both provided by the manufacturer). Coupling gel with a gel pad was placed on the skin for stand-off scanning. Contrast pulse sequence (CPS) imaging mode was used for evaluation of tumor perfusion with mechanical index of 0.25, frame rate of 5 Hz, dynamic range of 78 dB, and imaging depth of 3 cm. These settings were adjusted at the beginning and maintained constant during all of the experiments. For the ultrasound imaging studies, each mouse was anesthetized by i.p. injection of pentobarbital sodium (75 mg/kg, Sigma, USA). All ultrasound examinations were performed by one radiologist (J.H.Z., with 7 years experience in contrast-enhanced ultrasound), who was blind to the treatment status. Before contrast agent injection, the greatest longitudinal, transverse, and anteroposterior dimensions of tumors were measured in grayscale imaging using calipers. Tumor volume was calculated using the formula for a prolate ellipsoid: volume = π/6 × length × width × depth. The image plane containing the tumor at its maximum cross section and a large portion of the right lobe of the liver was imaged with the transducer held manually in this position throughout the examination.

Microbubble-based contrast agent SonoVue (Bracco, Milan, Italy) was used for contrast-enhanced ultrasound imaging. It is a sulfur hexafluoride gas containing agent with a phospholipids membrane. SonoVue was dissolved with physiologic saline to 5 ml and was injected as a bolus (0.1 ml/20 g) through the retroorbital vein with a 27-gauge needle. The bolus injection was performed by one radiologist (W.Z., with 5 years experience in small animals study) within 1sec for all animals to minimize variations of injection technique. Imaging was recorded on cine clips starting just before the contrast agent injection and continuing for 60 sec.

For CEUS study, twenty nude mice were randomly divided into two groups (i.e., treatment group and control group, n = 10 on each). The 10 mice in treatment group were treated with adriamycin (Shenzhen Main Luck Pharmaceuticals Inc., Guangdong, China) by intraperitoneal injection (4 mg/kg) once daily. The other 10 mice in control group were received vehicle control medium (sterile saline) with same timing and dosing schedule used for treatment group. The time point for the first dose given was referred to as day 0. Ultrasound imaging was performed at days 0, 2, 4 and 6 before each dosing.

### Imaging Analysis

**Figure 1:**
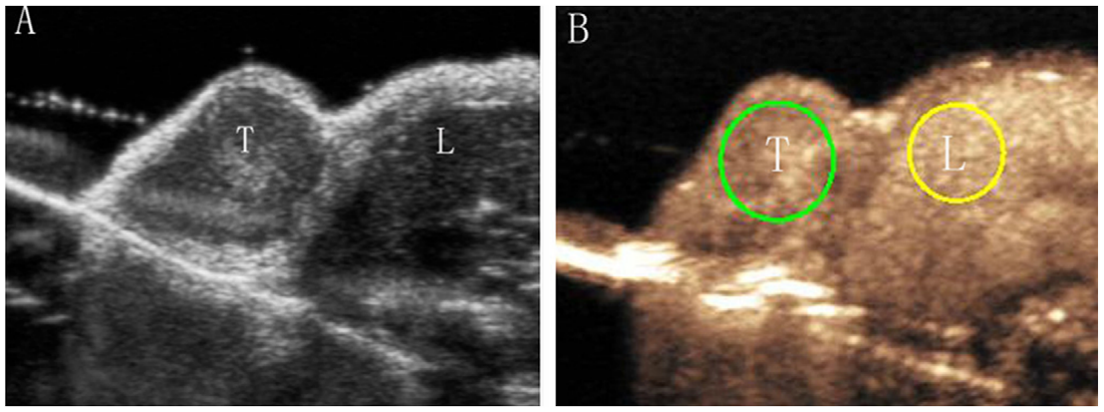
US image of a subcutaneous MCF7 breast cancer tumor. A, US scan showed gray-scale ultrasound image of tumor (T) adjacent to the right lobe of the liver (L). B, CEUS shows enhancement of normal liver parenchyma and tumor. Measurements obtained from one ROI drawn on the tumor (green ROI) and one drawn at matched depth in liver parenchyma (yellow ROI).

All datasets were transferred to a commercially available computer workstation (SonoTumor software, Bracco Research SA, Geneva, Switzerland). A region of interest (ROI) that drawn along the margin of the tumor and a ROI at matched depth in the region of the right lobe of the liver parenchyma were selected by one investigator (J.W.W., with 3 years experience in contrast-enhanced ultrasound) who was blind to the treatment information (Figure 1). The selected ROI was automatically positioned by the software on subsequent images, with minor adjustments to correct for respiratory motion when necessary. The analysis applies first linearization at the pixel level to revert the effects of “log” compression in the ultrasound system. Results obtained from the selected ROI represented an approximately linear depiction of the backscattered intensity. The average of the linearized intensities of all the pixels in the ROI was calculated to produce a time-signal intensity curve, where the intensity is theoretically linked to microbubble concentration [13].

Perfusion parameters of the tumor, including peak enhancement (PE), area under the curve of wash-in (WiAUC), wash-in rate (WiR) and wash-in perfusion index (WiPI) were calculated and normalized to those of the depth-matched liver parenchyma (Perfusion parameters _tumor_/Perfusion parameters _liver_). The results were noted as nPE, nWiAUC, nWiR and nWiPI (n = normalized). PE was defined as the maximum intensity of the signal produced by injection of the contrast agent. WiAUC was defined as the area under the curve from staring enhancement to peak enhancement. WiR was defined as the maximum slope between the time of onset of contrast inflow and the time of peak enhancement. WiPI was defined as area under the curve of wash-in divided by rise time. Quality of fit was used to test the fit between the raw data and the fitted mathematic model.

### Histopathologic Analysis

A separate group of mice with MCF-7 tumors were prepared (n = 40) and divided into control and adriamycin-treated (4 mg/kg, i.p.) groups. Mice were treated identically to those entered into the imaging studies above. Mice (n = 5 per time point) from the control and treated groups were sacrificed and tumors were excised on days 0, 2, 4 and 6. One minute before mice were sacrificed, Hoecst 33342 (25 mg/kg in water, Sigma-Aldrich Co., St. Louis, MO) was injected via the retroorbital vein. Tumors were rapidly excised and cut in half along a plane approximating to the imaging plane. One half of each tumor was snap frozen over liquid nitrogen for fluorescence microscopy, and one half was fixed in 10% buffered formalin before paraffin processing. Cryostat sections (five-micrometer-thick) were cut and Hoechst 33342 labeled cells were visualized and photographed under a fluorescence microscope equipped with a camera with 365 nm excitation and 420 nm emission filters showing blue fluorescence. Five different high power fields (×400) were randomly chosen within the highest fluorescence points and Image pro plus software (image pro-plus 6.0, Media Cybernetics, Silver Spring, MD, USA) was used to calculate the number of Hoechst 33342 labeled cells. Hoechst 33342 is a dye that stains the nuclei of endothelial cells, lining blood vessels that are perfused at the time of injection, and thus affords a measure of functional tumor vasculature. The mean number of Hoechst 33342 labeled cells reflected the blood perfusion of tumor [14].

Four-µm-thick sections were stained with hematoxylineosin (H&E) to assess tumor cell morphology changes. Immunohistochemical analysis was used to assess endothelial cell density (CD34-stained) and proliferating cell density (Ki67-stained). Antigen-retrieval procedure using citrate acid (pH of 6.0) was performed. Following heat-induced epitope retrieval in citrate buffer (pH of 6.0), the following primary antibodies were used, including rat antimouse CD34 antibody (clone MEC14.7, Abcam, UK) at 1∶100 dilution and mouse anti-human Ki67 antibody (clone MIB-1; Dako, Glostrup, Denmark) at 1∶50 dilution. The slides for evaluating CD34 expression were then incubated with a secondary rabbit antirat antibody (Zhongshan Goldenbridge Biology, Beijing, China) and those for evaluating Ki67 expression were then incubated with a secondary goat antimouse antibody (Zhongshan Goldenbridge Biology, Beijing, China). Also, Diaminobenzidine (DAB) was used for color development.

Regions with the highest tumor cell density in H&E stained sections were located by scanning the tissue sections under ×40-power microscope. After identification of the regions of highest tumor cell density, ten different fields were randomly chosen within the regions of highest tumor cell density at ×400 powers. The histopathologic images of each ×400 powers field were saved as a jpeg format in the computer for the measurement of tumor cell density as the number of nucleus in each image. Image pro plus software was used to calculate the number of nucleus in each image. The average of ten 400 powers field results was used for statistical analysis.

The measurements of MVD by counting the CD34-stained vessels under light microscopy were performed independently by two pathologists (with 5 and 10 years MVD working experience, respectively), who were blinded to the tumor treatment and ultrasound findings. According to the method by Weidner et al [15], regions with the highest vessel density (“hot spots”) were located by scanning the tissue sections under ×40-power microscope. After identification of the “hot spots”, three different fields were randomly chosen within each hot spot, and each endothelial cell or cell cluster that showed antibody staining and that was clearly separated from adjacent clusters was counted at ×200 powers for MVD measurements. The average of the two observers’ results was used for statistical analysis.

The proliferating cell density of the Ki-67 staining was defined as the average number of Ki-67 staining nuclei in 10 random fields (including periphery and centre of specimen) according to the method described by Dowsett [16]. Ten different fields were randomly chosen at ×400 powers and corresponding 10 pictures were taken in each tumor slice. Again, Image pro plus software was used to calculate the number of Ki-67 staining nuclei in each 400 powers field image. The average of ten 400 powers field results was used for statistical analysis.

### Statistical Analysis

All statistical analyses were performed by SPSS version 16.0 (SPSS, Inc, Chicago, IL). Independent samples *t*-test was used to analyze the significant differences of tumor volume, perfusion parameters, tumor perfusion (Hoechst 333342 staining), tumor cell density, proliferating cell density and MVD between the treatment and control groups. Paired-samples *t*-test was used to compare the perfusion parameters measured longitudinally by CEUS. Differences between baseline and follow-up histopathologic results were explored with the use of the Bonferroni corrected *t* test. The Pearson correlation test was used to examine the relationship between mean changes in the perfusion parameters measured by CEUS with tumor volume change. A *p* value of 0.05 or less was considered statistically significant.

## Results

### Effect of Adriamycin on Tumor Growth

**Figure 2:**
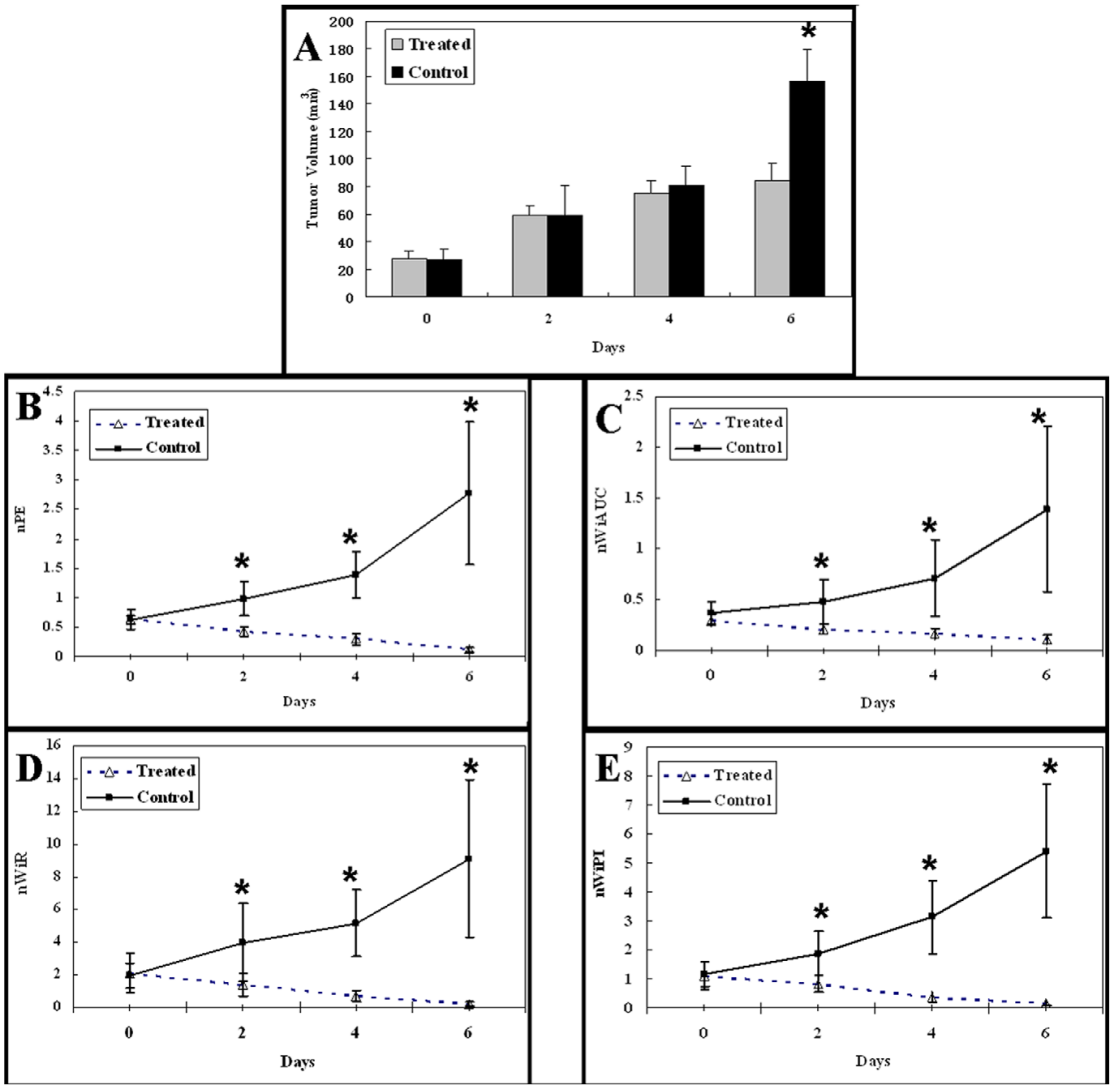
Changes of tumor volume and normalized perfusion parameters. A, There were significant differences of tumor volumes between control and treated groups on days 6 (* = *P*<0.001) while there were no significant differences in tumor volume on days 0, 2 and 4 (*P*>0.05). B-E, Changes in nPE (B), nWiAUC (C), nWiR (D) and nWiPI (E) at the different time points studied. CEUS demonstrated that all four normalized perfusion parameters of the tumors in the control group increased on days 2, 4 and 6, while in the treatment group, all four normalized perfusion parameters of the tumors decreased as early as 2 days after adriamycin therapy and remained low throughout the entire observation period. There were significant differences in the four normalized perfusion parameters between control and treatment groups on day 2, 4 and 6 (* = *P<*0.01).

Before therapy, there was no significant difference in tumor volumes between control and treatment groups (*P* = 0.795). The tumor volumes in two groups increased gradually on days 2 and 4, but no significant differences were seen between two groups at these time points (*P* was 0.970 and 0.275, respectively). On day 6, the tumor volume was significantly lower in the treatment group than the control group (the mean tumor volumes from control group = 156.44±22.58 mm^3^, from treatment group = 84.74±11.59 mm^3^, *P<*0.001). These results are illustrated in Figure 2A.

### Early Effect of Adriamycin on Tumor Perfusion Measured on CEUS

There were no significant differences in 4 normalized perfusion parameters (i.e., nPE, nWiAUC, nWiR and nWiPI) between control and treatment groups before treatment (*P*>0.05). In the control group, CEUS demonstrated that all four normalized perfusion parameters of the tumors significantly increased on day 2, 4 and 6 as compared with day 0 (*P<*0.05), while in the treatment group, all four normalized perfusion parameters of the tumors significantly decreased as early as 2 days after adriamycin therapy and remained low throughout the entire observation period as compared with day 0 (*P<*0.05). There were significant differences in the four normalized perfusion parameters between control and treatment groups on days 2, 4 and 6 (*P<*0.01) (Figure 2B, 2C, 2D and 2E). It was noticed that CEUS demonstrated a reduction of tumor perfusion 2 days after treatment which was 4 days earlier before the difference of tumor sizes became measurable by conventional imaging. At day 6, changes in the four normalized perfusion parameters measured by CEUS well correlated with the tumor volume change (for nPE, r = 0.702, *P* = 0.001; for nWiAUC, r = 0.534, *P* = 0.015; for nWiR, r = 0.759, *P*<0.001; for nWiPI, r = 0.732, *P*<0.001).

### Early Effect of Adriamycin on Tumor Perfusion (Hoechst 33342 Staining)

**Figure 3:**
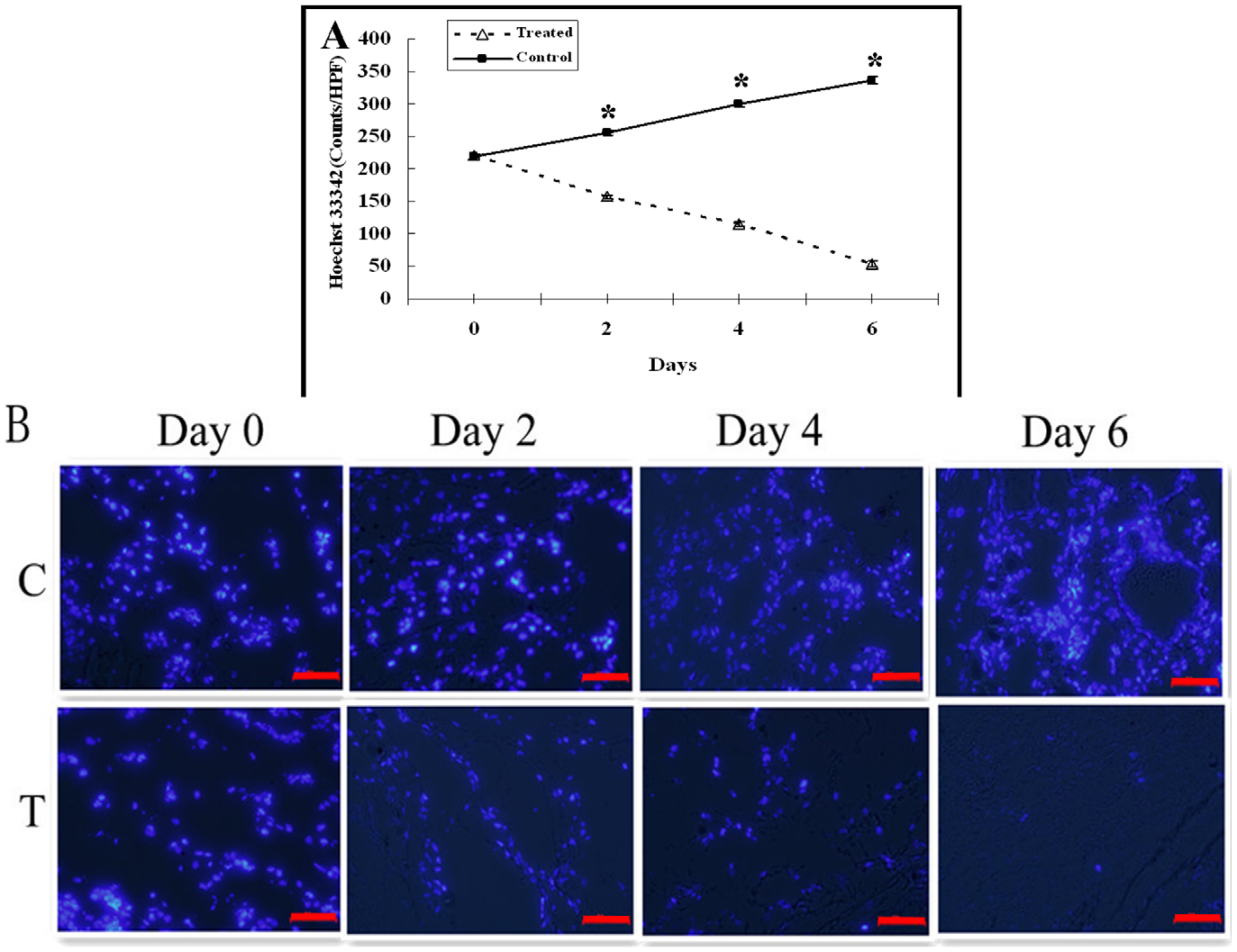
Histopathologic analysis of tumor perfusion changes (Hoechst 333342 staining). A, The graph showed the changes of tumor perfusion post treatment. There was significant difference in the number of Hoechst 33342 labeled cells between control and treatment groups on days 2, 4 and 6 (* = *P<*0.01). B, Representative Hoechst 333342 stained sections of the control (C) and treated (T) tumors on days 0, 2, 4 and 6. Scale bars: 50 µm.

The number of Hoechst 33342 labeled cells reflected the blood perfusion of tumor. There was no significant difference in the number of Hoechst 33342 labeled cells between control and treatment groups before treatment (*P* = 0.578). In the control group, Hoechst 33342 labeled cells significantly increased on day 2, 4 and 6 as compared with day 0 (*P<*0.001), while in the treatment group, Hoechst 33342 labeled cells significantly decreased on day 2, 4 and 6 as compared with day 0 (*P<*0.001). There was significant difference in the number of Hoechst 33342 labeled cells between control and treatment groups on days 2, 4 and 6 (*P<*0.01) (Figure 3).

### Effect of Adriamycin on Tumor on Cell Density

**Figure 4:**
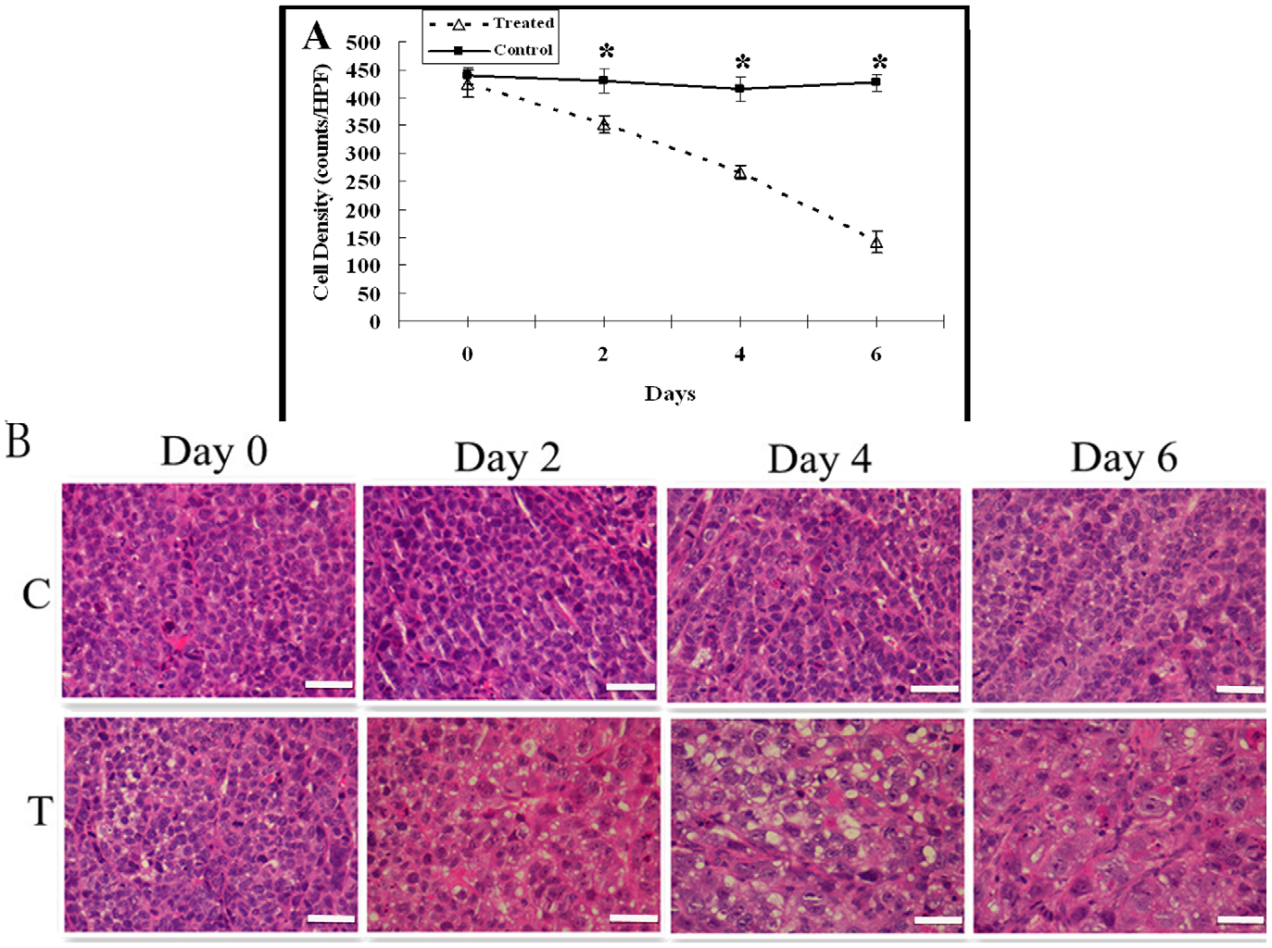
Histopathologic analysis of tumor cell density changes. A, The graph shows the changes of mean tumor cell density of the control and treated tumors post treatment. There was significant difference in tumor cell density between control and treatment groups on days 2, 4 and 6 (* = *P*<.001). B, Representative photomicrographs of hemotoxylin and eosin stained sections of the control (C) and treated (T) tumors on days 0, 2, 4 and 6. Scale bars: 50 µm.

There was no significant difference in the tumor cell density between control and treatment groups before treatment (*P* = 0.298). In the control group, tumor cell density remained stable on day 2, 4 and 6 as compared with day 0 (*P>*0.05), while in the treatment group, tumor cell density significantly decreased on day 2, 4 and 6 as compared with day 0 (*P<*0.001). There was significant difference in tumor cell density between control and treatment groups on days 2, 4 and 6 (*P<*0.001). (Figure 4).

### Effect of Adriamycin on Tumor Cell Proliferating

**Figure 5:**
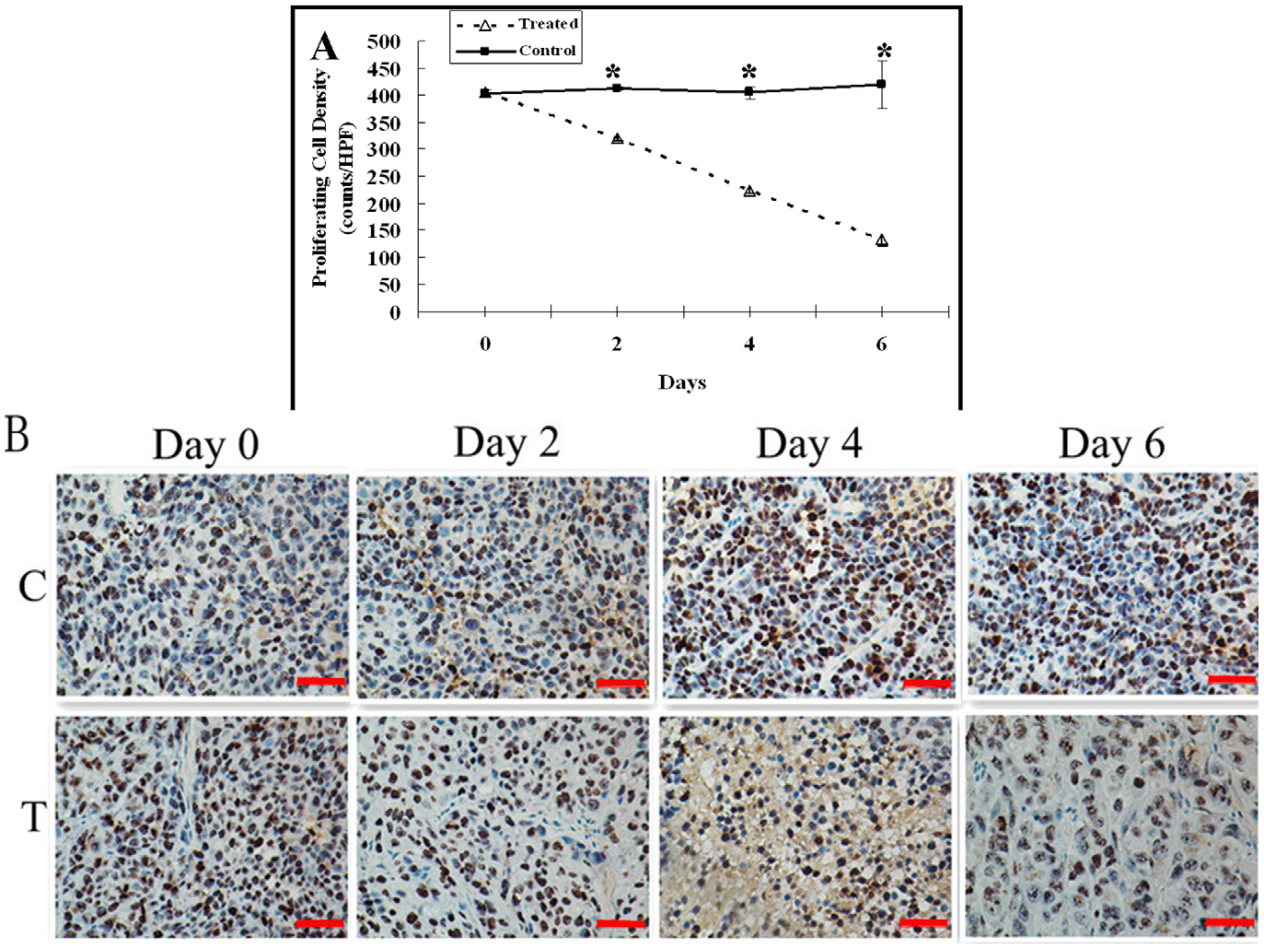
Immunohistochemical analysis of proliferating cell density changes. A, The graph shows the changes of mean proliferating cell density of the control and treated tumors post treatment. There was significant difference in proliferating cell density between control and treatment groups on days 2, 4 and 6 (* = *P*<0.001). B, Representative KI67-stained sections of the control (C) and treated (T) tumors on days 0, 2, 4 and 6. Brown areas reflect positive staining of proliferating tumor cell. Scale bars: 50 µm.

There was no significant difference in the proliferating cell density between control and treatment groups before treatment (*P* = 0.69). In the control group, tumor proliferating cell density remained stable on day 2, 4 and 6 as compared with day 0 (*P>*0.05), while in the treatment group, proliferating cell density significantly decreased on day 2, 4 and 6 as compared with day 0 (*P<*0.001). There was significant difference in proliferating cell density between control and treatment groups on days 2, 4 and 6 (*P<*0.01). (Figure 5).

### Effect of Adriamycin on Tumor MVD

**Figure 6:**
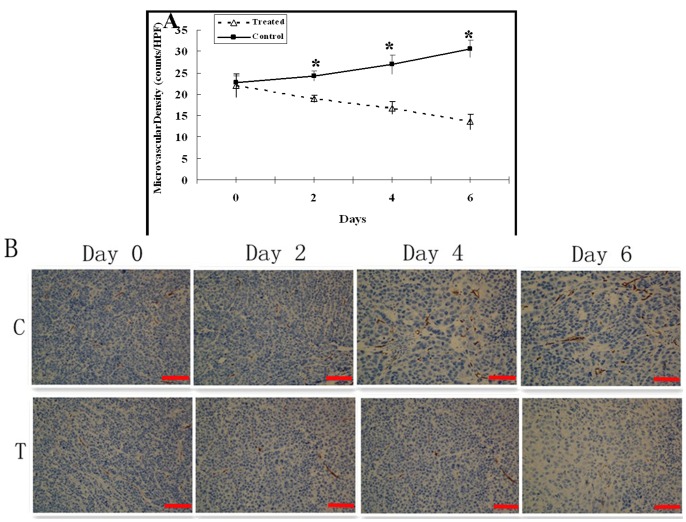
Immunohistochemical analysis of tumor MVD changes. A, The graph shows the changes of mean MVD in the control and treated tumors post treatment. There was significant difference in MVD between control and treatment groups on days 2, 4 and 6 (* = *P*<0.001). B, Representative CD34-stained sections of the control (C) and treated (T) tumors on days 0, 2, 4 and 6. Brown areas reflect positive staining of endothelial cells. Scale bars: 100 µm.

There was no significant difference in the tumor microvascular density (MVD) determined by immunohistochemical evaluation of endothelial cell (CD34) density between control and treatment groups before treatment (*P* = 0.635). In the control group, MVD increased from day 0 to day 2 although not significantly difference (*P* = 0.106) and then significantly increased on days 4 and 6 as compared with day 0 (*P<*0.05), while in the treatment group, MVD decreased from day 0 to day 2 although not significantly difference (*P* = 0.135), and then significantly decreased on days 4 and 6 as compared with day 0 (*P<*0.05). There was significant difference in MVD between control and treatment groups on days 2, 4 and 6 (*P<*0.001). (Figure 6).

## Discussion

Therapeutic effectiveness of adriamycin was detected as early as 2 days after treatment by using CEUS imaging to quantify tumor perfusion changes. In contrast, the measurements of the tumor volumes after therapy were not significantly different up to 6 days, implying that it is not feasible to assess early response to chemotherapy by monitoring tumor dimensions, as is standard in most clinical surveillance of the tumor treatment using RECIST protocol [2]. The present study demonstrated the feasibility and possibility of quantitative CEUS technique in predicting early response of breast cancer to cytotoxic chemotherapy in an animal model. The preliminary results shown that the decrease in blood perfusion of treated tumors on CEUS was associated with reduction in tumor perfusion (Hoechst 333342 staining), tumor cell density, proliferating cell density and MVD, as shown by both histopathologic and immunochemistry examinations in this study.

Traditionally, clinical examination and conventional imaging techniques, such as CT and MRI have been used to assess tumor size and response to the treatment. However, many studies have shown that clinical examination and conventional imaging are inappropriate techniques since assessing the tumor size by morphological measurement could result in over- or under-estimating the tumor response to chemotherapy [17], [18]. In addition, some tumor types are not easily measured because of their growth behavior. For example, malignant pleural mesothelioma grows as a ‘rind’ of tissue arising from the pleura rather than by discrete nodules, so that measuring the long axis is not appropriate [19]. Furthermore, both inter-observer and intra-observer errors often occur for a number of reasons such as poorly defined lesions, irregular shape lesions and when imaging occurs at different phases of contrast enhancement [1]. Therefore, it has been well recognized that tumor size change is an imperfect assessment method for assessing the effects of chemotherapy.

Reduction in tumor size is only a late sign of effective chemotherapy, so that the early response of tumors to therapy is difficult to assess using conventional radiographic modalities [20]. Early assessment of therapeutic efficacy is critical to prevent unnecessary treatment and optimize therapeutic strategies in order to obtain the optimal outcome of the therapy. Since it is very important for assessing early tumor response to cytotoxic chemotherapy, several functional imaging modalities, such as PET), DCE-MRI and DCE-CT, are being investigated to determine their ability to detect the tumor response in its early stage of the treatment, and hence facilitate the tailoring of treatment to response. PET with (18F) fluorodeoxyglucose (FDG) is widely used for clinical staging and treatment evaluation of cancer by depicting a reduction in the metabolic activity of the tumor [21]. Although FDG PET has higher sensitivity than anatomic imaging modalities, it suffers from low spatial resolution and exposes patients to ionizing radiation (as does CT). The extent of FDG uptake by lesions smaller than twice the quoted resolution of PET is often underestimated [22]. Both DCE-MRI and DCE-CT have shown potentials as functional imaging tools to early assess tumor response to chemotherapy by detecting tumor blood perfusion changes [5], [23]–[25]. Although DCE-MRI offers relatively good sensitivity and spatial resolution in soft tissue imaging, the spatial resolution must be traded off to get a sufficient frame rate for dynamic tracking of the contrast agent in order to perform whole organ scans with functional imaging information [26]. Although DCE-CT imaging is suitable for assessment of tissue perfusion [26], high concentration of CT contrast agent together with the relatively high dose of radiation limit the utilization of CT for frequent evaluation of tumor response to therapy.

Ultrasound imaging has been frequently used in clinical practice for monitoring effects of tumor therapy. Since color and spectral Doppler ultrasound allows semiquantitative assessment of tumor vascularity, measurements of peak systolic velocity, end-diastolic velocity, resistivity index and pulsatility index have shown the potential to serve as a functional tool for monitoring tumor response to chemotherapy [27]–[29]. However, the high degree of subjectivity as well as its lack of reproducibility makes it impossible to gain wide acceptance in clinical practice [30]. With the wide application of ultrasound contrast agents in clinical practice, CEUS has recently improved the US diagnostics of several diseases, especially in the differentiation of liver lesions [31]. CEUS offers great advantages of evaluating tumor perfusion in real time compared to other methods for assessing tumor blood perfusion. Quantitative evaluation of tumor perfusion with CEUS has been used to evaluate tumor response to antiangiogenic agents which work by typically preventing the development of new blood vessels needed to support tumor growth both in animal [8]–[11] and clinical [32], [33] studies, and preliminary results were promising. In this study, the early treatment effect of a cytotoxic chemotherapeutic drug, adriamycin, was evaluated with CEUS for the first time, and decrease in tumor perfusion was detected by CEUS in treated tumors as early as 2 days after therapy, whereas measurable tumor shrinkage with significance difference in the volume was noticed 6 days after treatment, which gives a great potential for this technique to assess early tumor response to therapy in future preclinical and clinical studies.

The pathophysiologic basis for the tumor perfusion changes seen in this study after chemotherapy is not yet fully understood, but is likely to be multifactorial, relating to changes in both requirement for blood supply and microvessel changes. Adriamycin, an anthracyclinic antibiotic, is a DNA-interacting drug for treatment of various types of cancers, such as ovarian, breast, prostate, cervix and lung cancers. In the present study, the tumor cell density and proliferating cell density of treated tumors was significantly less than those of control tumors. The death of cancer cells and inhibition of proliferating activities caused by adriamycin induced the reduction of tumor metabolic activity, which will lead to the decrease of tumor blood supply. On the other hand, it has been suggested that successful chemotherapy causes cytotoxic tumor cell death resulting in a reduction in tissue vascular endothelial growth factor levels and hence apoptosis of immature endothelial cells with secondary vascular shutdown [34]. Furthermore, most of the conventional chemotherapy drugs do not only damage the tumor cells but also the endothelial cells [35], but also contribute to the decrease of tumor perfusion. In this study, we found that Adriamycin-treated tumors were associated with a significant decrease of MVD as compared with control tumors.

There are some limitations in this study. First, a potential limitation of CEUS technique is that possible variations in contrast agent kinetics could occur through differences in the injection rate or other injection artifacts. However, in the case of a briefly injected 0.1 mL bolus, these variations should not lead to a significant bias as the injection was systematically performed by one investigator for all animals. Second, calculating tumor perfusion in a single tumor section may have introduced a bias since tumor tissue is inhomogenous in volume. A complete quantification of tumor perfusion throughout the whole tumor volume could improve the relevance of the tumor perfusion parameters. Third, although xenografted tumors are well-accepted and important experimental model systems, they obviously do not mimic human tumor behavior exactly and a single cancer cell line might not be representative of the different types of human breast cancer. Further clinical evaluation is necessary to confirm the validity of quantitative CEUS on early assessment of human tumors response to chemotherapy. Finally, the use of a two-arm study design (control vs. treatment) limits the conclusions regarding the utility of CEUS. A better study design would be to correlate changes in CEUS parameters of tumors with the outcomes of individual animals.

In conclusion, this study indicates that quantitative CEUS provided a simple way to early monitor tumor response to cytotoxic chemotherapy by detecting tumor perfusion changes. Dynamic CEUS for quantification of tumor perfusion could be used for early detection of cancer response to cytotoxic chemotherapy prior to notable tumor shrinkage.
